# Antimicrobial use and resistance in *Escherichia coli* from healthy food-producing animals in Guadeloupe

**DOI:** 10.1186/s12917-021-02810-3

**Published:** 2021-03-08

**Authors:** Gaëlle Gruel, Arantxa Sellin, Hélène Riveiro, Matthieu Pot, Sébastien Breurec, Stéphanie Guyomard-Rabenirina, Antoine Talarmin, Séverine Ferdinand

**Affiliations:** 1 Laboratory of Microbial Ecosystems Interactions, Transmission Reservoir and Pathogens Diversity Unit, Institut Pasteur of Guadeloupe, Morne Joliviere - B.P. 484, 97183 Les Abymes Cedex, Guadeloupe France; 2 Faculté de Médecine Hyacinthe Bastaraud, Université des Antilles, Pointe-à-Pitre, Guadeloupe France; 3 Centre d’Investigation Clinique, INSERM CIC 1424, Pointe-à-Pitre, Guadeloupe France

## Abstract

**Background:**

Selection pressure exerted by use of antibiotics in both human and veterinary medicine is responsible for increasing antimicrobial resistance (AMR). The objectives of this study were to better understand antimicrobial use in pigs, beef cattle, and poultry on farms on Guadeloupe, French West Indies, and to acquire data on AMR in *Escherichia coli* in these food-producing animals. A cross-sectional survey was conducted at 45 farms on Guadeloupe, and practical use of antimicrobials was documented in declarative interviews between March and July 2018. A total of 216 fecal samples were collected between January 2018 and May 2019, comprising 124 from pigs, 75 from beef cattle, and 17 from poultry litter. *E. coli* isolates were obtained for further testing by isolation and identification from field samples. Antimicrobial susceptibility testing and screening for *bla*_CTX-M_, *bla*_TEM_, *tet*A, and *tet*B resistance genes by polymerase chain reaction on extracted genomic DNA were performed.

**Results:**

The study showed rational use of antimicrobials, consisting of occasional use for curative treatment by veterinary prescription. Tetracycline was the most commonly used antimicrobial, but its use was not correlated to *E. coli* resistance. Extended-spectrum β-lactamase (ESBL) *E. coli* isolates were detected in 7.3% of pigs, 14.7% of beef cattle, and 35.3% of poultry. *bla*_CTX-M-1_ was the predominant gene found in ESBL-*E. coli* isolates (68.8%), followed by *bla*_CTX-M-15_ (31.3%).

**Conclusion:**

Despite rational use of antimicrobials, the rate of ESBL-*E. coli* in food-producing animals in Guadeloupe, although moderate, is a concern. Further studies are in progress to better define the genetic background of the ESBL-*E. coli* isolates.

**Graphical abstract:**

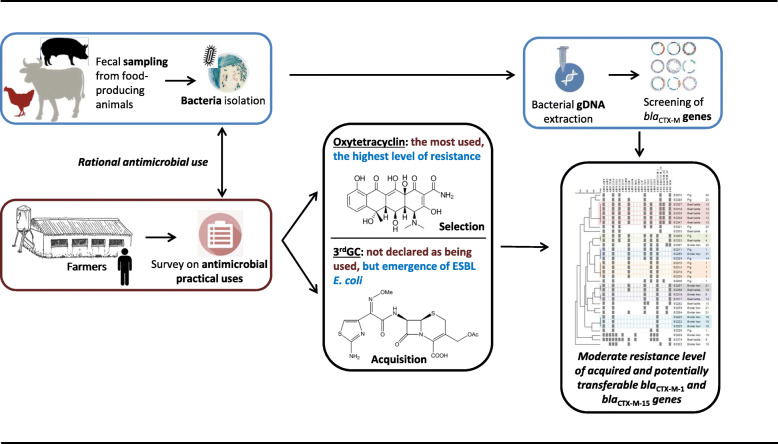

## Background

Currently, antimicrobial resistance (AMR) is one of the most urgent public health problems in the world [1]. It has dramatically increased morbidity and mortality in both humans and animals, with serious repercussions for future treatment of infections in humans and for animal health and productivity [2]. Administration of antimicrobials to animals is considered to be a major contributor to the emergence of AMR worldwide [3], and several high-income countries report extensive use of antimicrobials and AMR in animals [4]. To reduce the use of antibiotics in animals, the risk factors for infectious diseases, such as the genetic background of breeds and the management of farms, must be addressed [5].

Bacterial strain diversity has played a key role in the global emergence of AMR, and selection pressure by antibiotics imbalances diversity in favor of pathogens and greater resistance [6]. The emergence of extended-spectrum β-lactamase (ESBL)-producing Enterobacteriaceae, which hydrolyze key antimicrobials, such as the expanded-spectrum cephalosporins cefotaxime, ceftriaxone, ceftazidime, and cefepime, is due mainly to the selective pressure of antibiotics used in both human and veterinary medicine [7]. In 2014, the antimicrobial consumption was higher in animals (152 mg of active substance per kg of estimated biomass) than in humans (124 mg/kg) in Europe. Consumption of 3rd- and 4th-generation cephalosporins was associated with resistance in *E. coli* in humans. Tetracyclines and polymyxins resistance in *E. coli* from animals was associated with corresponding antimicrobial consumption in animals [8]. The resistance is mediated mainly by acquired ESBL genes located on mobile genetic elements and is frequently associated with resistance genes against several families of antimicrobial agents [9].

Guadeloupe, a French overseas department in the Caribbean, has been classified as a very high-resource country [10]. Less than one third of the surface of this small island is devoted to agriculture, and the livestock in 2018 comprised 14,500 pigs, 44,900 cattle, and 507,000 laying hens and broilers [11]. The latest of the few studies on AMR on Guadeloupe showed a low prevalence of ESBL-producing Enterobacteriaceae in human community-acquired urinary tract infections [12] and in wastewater treatment plants [13]. As there is close contact between humans and domestic animals, the animals may be reservoirs of resistance genes; however, the only data on antibiotic resistance in local domestic animals is a study on horses, which showed the emergence of various ESBL-producing *E. coli* clones, some of which persisted for more than a month after antibiotic treatment [14].

The main objective of this work was to obtain information on antimicrobial use on farms on Guadeloupe and on AMR levels in the zoonotic indicator bacteria *E. coli* in pigs, beef cattle, and poultry.

## Results

In our survey of use of medication containing antibiotics in pigs, beef cattle, and poultry on Guadeloupe, 64.4% (29/45) of all farmers reported their use during the last year (Table 1). Beef cattle were individually treated, whereas collective treatments to the entire flock were administrated in pig and poultry farms. Antimicrobials were usually administered as curative treatment (20/29, 69.0%) and under veterinary prescription (22/29, 75.9%). The main causes for which antimicrobials were given were respiratory diseases in pigs (5/11, 45.5%), skin diseases in cattle (5/12, 41.7%), and respiratory and digestive diseases in poultry (4/6, 66.7%) (Table 1).

**Table 1:** Use of antimicrobials in poultry, pig, and beef cattle production systems on Guadeloupe

	Farms								
	Poultry		Pig		Beef cattle		Total		*P*
	(*n* = 15)		(*n* = 14)		(*n* = 16)		(*n* = 45)		
**Herd size**									
mean *± sd*	12,301	*(18,983.0)*	611	*(799.0)*	106	*(197.0)*	4327.8	*(12,141.0)*	
median *(iqr)*	1500	*(19,340.0)*	445	*(603.0)*	36	*(50.0)*	400.0	*(948.0)*	**< 0.001**
**Use of food supplements**^***a***^ **n,** ***(%)***									
*Never*	1	*(6.7)*	5	*(35.7)*	6	*(35.7)*	12	*(26.7)*	
*Occasionally*	5	*(33.3)*	6	*(42.9)*	2	*(12.5)*	13	*(28.9)*	NS
*Systematically*	9	*(60.0)*	3	*(21.4)*	8	*(50.0)*	20	*(44.4)*	
**Use of antimicrobial agent**									
*Never*	9	*(60.0)*	3	*(21.4)*	4	*(25.0)*	16	*(35.6)*	
*Occasionally*	4	*(26.7)*	10	*(71.4)*	12	*(75.0)*	26	*(57.7)*	**0.046**
*Systematically*	2	*(13.3)*	1	*(7.1)*	0	*(0.0)*	3	*(6.7)*	
**Veterinarian is the drug supplier**^***d***^									
*yes*	6	*(100.0)*	8	*(72.7)*	8	*(66.7)*	22	*(75.9)*	NS
*Unknown*	0	*(0.0)*	3	*(27.3)*	4	*(33.3)*	7	*(24.1)*	
**Nature of antimicrobial treatment**									
*Preventive*	3	*(50.0)*	5	*(45.5)*	1	*(8.3)*	9	*(31.0)*	**0.027**
*Curative*	3	*(50.0)*	6	*(54.5)*	11	*(91.7)*	20	*(69.0)*	
**Reasons for treatment**^***b***^									
*Skin disease*	0	*(0.0)*	1	*(9.1)*	4	*(33.3)*	5	*(17.2)*	
*Respiratory pathology*	1	*(16.7)*	1	*(9.1)*	0	*(0.0)*	2	*(6.9)*	
*Skin disease and other*^*c*^	0	*(0.0)*	1	*(9.1)*	1	*(8.3)*	2	*(6.9)*	
*Respiratory and digestive pathologies*	1	*(16.7)*	1	*(9.1)*	0	*(0.0)*	2	*(6.9)*	
*Respiratory, digestive pathologies and other*^*c*^	1	*(16.7)*	1	*(9.1)*	0	*(0.0)*	2	*(6.9)*	NS
*Digestive pathology*	1	*(16.7)*	0	*(0.0)*	0	*(0.0)*	1	*(3.4)*	
*Skin disease and respiratory pathology*	0	*(0.0)*	1	*(9.1)*	0	*(0.0)*	1	*(3.4)*	
*Skin disease, respiratory and digestive pathologies*	0	*(0.0)*	1	*(9.1)*	0	*(0.0)*	1	*(3.4)*	
*Digestive pathology and other*^*c*^	0	*(0.0)*	0	*(0.0)*	1	*(8.3)*	1	*(3.4)*	
*Other*^*c*^	1	*(16.7)*	4	*(36.3)*	5	*(41.8)*	10	*(34.5)*	
*Unknown*	1	*(16.7)*	0	*(0.0)*	1	*(8.3)*	2	*(6.9)*	
**Antimicrobials used**									
*Tetracyclines*	1	*(16.7)*	4	*(36.3)*	9	*(75.1)*	14	*(48.4)*	**0.001**
*Trimethoprim-sulfamethoxazole*	4	*(66.6)*	0	*(0.0)*	0	*(0.0)*	4	*(13.9)*	
*β-lactams + streptomycin + tetracyclines*	0	*(0.0)*	2	*(18.2)*	1	*(8.3)*	3	*(10.4)*	
*β-lactams + streptomycin*	0	*(0.0)*	2	*(18.2)*	0	*(0.0)*	2	*(6.9)*	
*Tetracyclines + trimethoprim-sulfamethoxazole*	1	*(16.7)*	0	*–*	0	*(0.0)*	1	*(3.4)*	
*β-lactams*	0	*(0.0)*	1	*(9.1)*	0	*(0.0)*	1	*(3.4)*	
*β-lactams + tetracyclines*	0	*(0.0)*	0	*(0.0)*	1	*(8.3)*	1	*(3.4)*	
*β-lactams + phenicols + colistin + macrolides*	0	*(0.0)*	1	*(9.1)*	0	*(0.0)*	1	*(3.4)*	
*Streptomycin + tetracyclines*	0	*(0.0)*	0	*(0.0)*	1	*(8.3)*	1	*(3.4)*	
*Streptomycin + phenicols + macrolides*	0	*(0.0)*	1	*(9.1)*	0	*(0.0)*	1	*(3.4)*	
*> 1 antimicrobial molecule*									
*no*	5	*(33.3)*	5	*(45.5)*	9	*(75.0)*	19	*(65.5)*	NS
*yes*	1	*(6.7)*	6	*(54.5)*	3	*(25.0)*	10	*(34.5)*	
**Route of administration**^***d***^									
*Parenteral*	0	*(0.0)*	8	*(72.7)*	11	*(91.7)*	19	*(65.5)*	**< 0.0001**
*Feed*	0	*(0.0)*	1	*(9.1)*	0	*(0.0)*	7	*(24.2)*	
*Oral*	6	*(100.0)*	1	*(9.1)*	0	*(0.0)*	1	*(3.4)*	
*Unknown*	0	*(0.0)*	1	*(9.1)*	1	*(8.3)*	2	*(6.9)*	
**One molecule used for 2 distinct pathologies**									
*yes*	2	*(33.3)*	1	*(9.1)*	1	*(8.3)*	4	*(13.8)*	NS
*no*	2	*(33.3)*	4	*(36.4)*	5	*(41.7)*	11	*(38.0)*	
**More than one molecule used for the same pathology**									
*yes*	1	*(16.7)*	2	*(18.1)*	2	*(16.7)*	5	*(17.2)*	NS
*no*	0	*(0.0)*	4	*(36.4)*	1	*(8.3)*	5	*(17.2)*	
*Unknown*	1	*(16.7)*	0	*(0.0)*	3	*(25.0)*	4	*(13.8)*	
**Vaccine administration**									
*yes*	2	*(13.3)*	4	*(28.6)*	0	*(0.0)*	6	*(13.3)*	NS
*no*	13	*(86.7)*	10	*(71.4)*	16	*(100.0)*	39	*(86.6)*	
**Other treatments**^***e***^									
*yes*	2	*(13.3)*	5	*(35.7)*	4	*(25.0)*	11	*(24.4)*	NS
*no*	13	*(86.7)*	9	*(64.3)*	12	*(75.0)*	34	*(75.6)*	
**Veterinary treatment cost estimation in €/100 kg/year**									
mean *± sd*	10.6	*(18.1)*	11.2	*(10.1)*	6.2	*(5.3)*	9.2	*(12.2)*	
median *(iqr)*	1.7	*(21.4)*	7.1	*(16.0)*	6.4	*(8.0)*	6.1	*(12.7)*	NS

Quantitative variables are summarized as median and interquartile range (IQR) or as mean ± standard deviation (SD). Qualitative variables are given as numbers and percentages. Intergroup differences were assessed in the Mann-Whitney test or chi-square test, as appropriate. Significant *P* values are shown in bold

*NS* not significant

^a^Trace elements, vitamins, carbohydrates, amino acids

^b^Treatments could be applied for more than one reason

^c^Infectious diseases, reproductive diseases, ticks, leg lesions

^d^Percentages were calculated with the number of farmers who declared use of antimicrobials as the denominator

^e^Antiparasitic, antihistaminic, hepatic, medicinal plants

The most commonly used active substance was tetracycline (20/29, 69.0%); β-lactams and streptomycin were administered by 27.6% (8/29) and 24.1% (7/29) of farmers, respectively. Among farmers who administered antibiotics, the proportion of tetracycline use was significantly higher in beef cattle (100.0%) than in pigs (54.5%) and poultry (33.3%). β-lactams were administered mainly by pig farmers (54.5%), and most of the poultry producers used only trimethoprim–sulfamethoxazole (83.3%, *P* <  0.001). Antimicrobials were used significantly less frequently in poultry (40.0%) than in pigs (78.6%) or beef cattle (75.0%) (*P* = 0.046). The median annual cost of veterinary treatment (drugs and veterinary fees) per 100 kg was estimated to be 7.2 € for pigs, 6.5 € for adults beef cattle, and 1.7 € for poultry.

A total of 216 fecal samples were collected from food-producing animals on 28 farms and in the slaughterhouse (34 additional farms). Samples were collected from 75 adult beef cattle for meat production (51 on farms and 24 at the slaughterhouse), 124 pigs included piglets, weaned, finishers, sows and breeding males (90 on farms and 34 at the slaughterhouse), and 17 hen houses representing 53,000 poultry. The prevalence of ESBL-*E. coli* was higher on poultry farms (4/9, 44.4%) than on beef cattle farms (4/32, 12.5%) or pig farms (3/21, 14.3%); however, the poultry samples were from litter, representing more than one bird (Table 2).

**Table 2:** Distribution of ESBL-*E. coli*-positive isolates

	Farms			Animals		
	Total	ESBL-*E. coli*		Total	ESBL-*E. coli*	
	(*n* = 62)	positive (*n* = 11)		(*n* = 216)	positive (*n* = 26)	
n, *(%)*						
**Beef cattle**	32	4	*(12.5)*	75	11	*(14.7)*
**Pig**	21	3	*(14.3)*	124	9	*(7.3)*
**Poultry**	9	4	*(44.4)*	17	6	*(35.3)*

*ESBL*, extended-spectrum β-lactamase producing isolated on selective plates

On the basis of the observed frequencies of AMR phenotypes, corresponding to ESBL-*E. coli* isolated on ceftriaxone selective plates and on non-selective plates for non-ESBL-*E. coli*, the highest levels of resistance were against ampicillin, cefotaxime, streptomycin, tetracycline, and trimethoprim–sulfamethoxazole. Significant differences in the frequencies of resistant *E. coli* isolates were found among the three production systems (Tables 1 and 3).

**Table 3:** Comparison of antimicrobial use and resistant *E. coli* frequencies in poultry, pigs, and beef cattle

	Farmers antimicrobial use					Resistant *E. coli* isolates								
	Poultry (*n* = 6)	Pig (*n* = 11)	Beef cattle (n = 16)	*P*		ESBL E. coli poultry (*n* = 10)	n-ESBL E. coli poultry (*n* = 13)	*P*	ESBL E. coli pigs (*n* = 11)	n-ESBL E. coli pigs (*n* = 146)	*P*	ESBL E. coli beef cattle (*n* = 11)	n-ESBL E. coli beef cattle (*n* = 74)	*P*
n, *(%)*					n, *(%)*									
TET	2 *(33.3)*	6 *(54.5)*	12 *(100.0)*	0.003	TET	5 *(50.0)*	8 *(61.5)*	*NS*	6 *(54.5)*	86 *(58.9)*	*NS*	9 *(81.8)*	6 *(8.1)*	< 0.001
β-lact^a^	0 *(0.0)*	6 *(54.5)*	2 *(16.7)*	0.035	AMP	10 *(100.0)*	8 *(61.5)*	*NS*	11 *(100.0)*	21 *(14.4)*	< 0.001	11 *(100.0)*	9 *(12.2)*	< 0.001
SM	0 *(0.0)*	5 *(45.5)*	2 *(16.7)*	NS	SM	4 *(40.0)*	6 *(46.2)*	*NS*	6 *(54.5)*	68 *(46.6)*	*NS*	8 *(72.7)*	22 *(29.7)*	0.025
SXT	5 *(83.3)*	0 *(0.0)*	0 *(0.0)*	< 0.001	SXT	3 *(30.0)*	5 *(38.5)*	*NS*	8 *(72.7)*	20 *(13.7)*	< 0.001	7 *(63.6)*	4 *(5.4)*	< 0.001
QN	0 *(0.0)*	0 *(0.0)*	0 *(0.0)*	NS	NAL	1 *(10.0)*	2 *(15.4)*	*NS*	0 *(0.0)*	3 *(2.1)*	*NS*	1 *(9.1)*	1 *(1.4)*	NS
C3G	0 *(0.0)*	0 *(0.0)*	0 *(0.0)*	NS	COX	10 *(100.0)*	0 *(0.0)*	< 0.001	11 *(100.0)*	0 *(0.0)*	< 0.001	11 *(100.0)*	0 *(0.0)*	< 0.001

*TET* tetracyclines, *SM* streptomycin, *SXT* trimethoprim–sulfamethoxazole, *QN* quinolones, *AMP* ampicillin, *NAL* nalidixic acid, *COX* cefotaxime

^a^Penicillins such as ampicillin and amoxicillin

We compared the rates of resistance to tetracycline, ampicillin, streptomycin, and trimethoprim–sulfamethoxazole according to the antibiotic or class of antibiotics reported in the survey, according to animal species. The number of farms in the ATB use survey is smaller than the number of farms investigated for fecal sampling. As mentioned above and shown in Table 1, although only one third of poultry farmers who used antibiotics used tetracycline as collective treatment, a high prevalence of tetracycline-resistant *E. coli* were found in poultry (Table 3). Use of β-lactams also did not correspond to the level of resistance to β-lactams in poultry (17/23, 73.9%), which did not receive these drugs. The proportion of tetracycline-, ampicillin-, cefotaxime-, streptomycin-, and trimethoprim–sulfamethoxazole-resistant ESBL-*E. coli* was similar to non-ESBL-*E. coli* from poultry (Table 3). ESBL-*E. coli* frequency was not associated to the rate of tetracycline and streptomycin resistance occurrence despite the use of these antimicrobials by half of the pig farmers. These resistances occurred independently of the detection of an ESBL. ESBL-*E. coli* frequency was associated to the rate of ampicillin (100.0% of ampicillin resistance in ESBL-*E. coli* vs 14.4 in non-ESBL-*E. coli*, *P* <  0.001) and trimethoprim–sulfamethoxazole resistance occurrence (72.7% of trimethoprim–sulfamethoxazole resistance in ESBL-*E. coli* vs 13.7%, in non-ESBL-*E. coli P* <  0.001), while β-lactams were used by half of the pig farmers and trimethoprim–sulfamethoxazole was not declared to be used. Regardless of the antimicrobials used, ESBL-*E. coli* isolates from beef cattle, individually treated, were significantly associated with other resistance carriage (*P* ≤ 0.025) (Table 3). Congruence was observed between the absence of quinolone use and a low frequency of nalidixic acid-resistant *E. coli* in the three animal species.

**Fig. 1:**
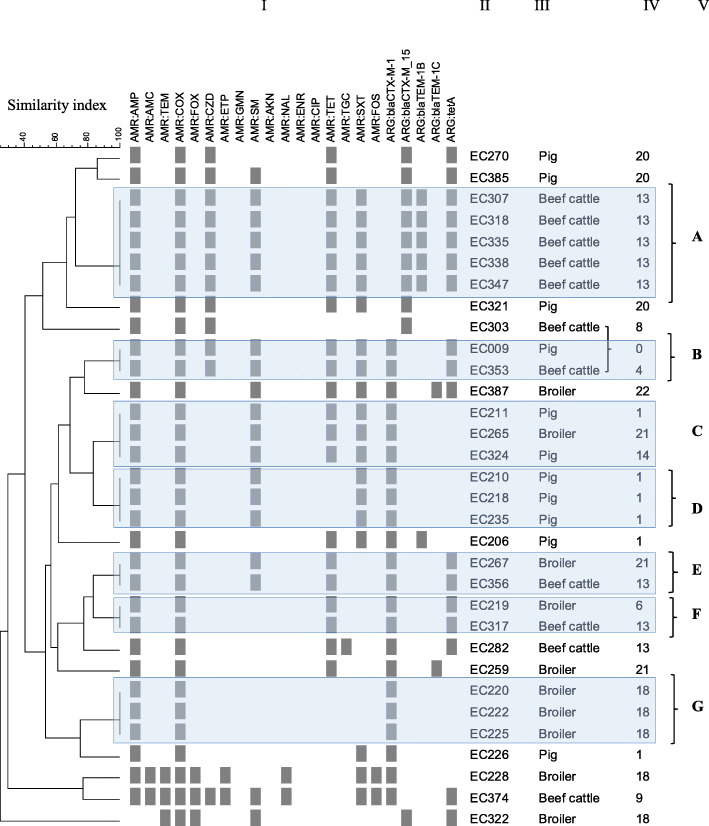
Combined numerical analysis of AMR and ARG patterns for 32 ESBL-*E. coli* isolates from food-producing animals in Guadeloupe. Patterns were based on AMR profile and *bla*_CTX-M_ and *bla*_TEM_ (ARG) gene screening. Seven clusters containing isolates from two to five samples can be seen. The bar represents the similarity index obtained by the unweighted pair group method with arithmetic averages. Column I, combined pattern of AMR and ARG; column II, isolate number; column III, sample origin; column IV, farm identifying, column V, cluster designation. AMR, antimicrobial resistance phenotype; ARG, antimicrobial resistance gene. AMP, ampicillin (10 μg); AMC, amoxicillin–clavulanic acid (20 μg–10 μg); COX, cefotaxime (5 μg); FOX, cefoxitin (30 μg); CZD, ceftazidime (10 μg); ETP, ertapenem (10 μg), GMN, gentamicin (10 μg); Sm, streptomycin (10 μg), AKN, amikacin (30 μg); NAL, nalidixic acid (30 μg); ENR, enrofloxacin (5 μg); CIP, ciprofloxacin (5 μg); TET, tetracycline (30 IU); TGC, tigecyclin (15 μg); SXT, trimethoprim–sulfamethoxazole (1.25 μg–23.75 μg); FOS, fosfomycin (200 μg)

The ESBL isolates harbored predominantly the *bla*_CTX-M-1_ gene (22/32, 68.8%), followed by *bla*_CTX-M-15_ (10/32, 31.3%) (Table 4). The *bla*_CTX-M-1_ gene was combined with the *bla*_TEM-1C_ in two pigs and with the *bla*_TEM-1B_ gene in one poultry isolate. The remaining ESBL-*E. coli* carried a *bla*_CTX-M-15_ gene, usually with combined cefotaximase and ceftazidimase activity. A comparison of phenotypic and genotypic profiles based on combined patterns analysis of AMR and antimicrobial resistance genes (ARG) is shown in Fig. 1. The *bla*_CTX-M_ genes were carried by ESBL-*E. coli* isolates found in the three food-producing animal systems on four farms in distinct geographic areas. The comparative analysis generated 18 distinct patterns of the 32 ESBL-*E. coli* (Fig. 1), with 20 (62.5%) isolates grouped into seven clusters with similar AMR/ARG patterns (A–G) comprising two to five isolates, whereas 12 (37.5%) distinct patterns were not clustered. Three clusters (A, D, G) included nine ESBL-*E. coli* at the same farm, whereas the other clustered ESBL-*E. coli* (B, C, E, F) were not specific to a production system. Twelve clustered isolates with similar AMR/ARG profiles were found in different food-producing farms (12/32, 37.5%); e.g. one cluster of ESBL-*E. coli* carriers (C) consisted of two pigs and one hen house on three different farms.

**Table 4:** Distribution of ESBL-*E. coli bla*_CTX-M_ gene type

	ESBL***-E. coli*** isolates			
	Total	*bla* carrier	*bla*_CTX-M-15_	*bla*_CTX-M-1_
	(*n* = 265)	(*n* = 32)	(n = 10)	(*n* = 22)
n, *(%)*				
**Beef cattle**	85	11 *(12.9)*	6 *(7.1)*	5 *(5.9)*
**Pig**	157	11 *(7.0)*	3 *(1.9)*	8 *(5.1)*
**Poultry**	23	10 *(43.5)*	1 *(4.3)*	9 *(39.1)*

*bla* CTX-M β-lactamase

## Discussion

This study of antimicrobial use on small-scale pig, beef cattle, and poultry farms in Guadeloupe showed moderate ESBL-*E. coli* in pig and beef cattle production, probably because of rational use of antimicrobials. The island adheres to the French AMR reduction plan [15] on the use of veterinary antimicrobials, and the moderate ESBL resistance may reflect its effectiveness. The frequencies were nevertheless higher than those in French national surveillance for AMR in infected animals in 2018, in which *E. coli* isolates resistant to third- and fourth-generation cephalosporins were detected in 2.3% of beef cattle and in < 2.0% of pigs, poultry, and turkeys [16]. The differences may be due to sampling of diseased rather than healthy animals for detection of ESBL-*E. coli.* The prevalence in our study is closer to that observed in Portugal, where 5.7% of fecal samples from 35 healthy pigs and 10% of those from healthy chickens were positive for ESBL-*E. coli* [17], but lower than that in Switzerland, where 15.3% of pigs and 13.7% of bovine fecal samples were positive [18]. In European countries, the occurrence of *E. coli* resistance in healthy animals at slaughterhouse varied from 0 to 7.9% in fattening pigs; from 0 to 5.9% in calves under 1 year of age [19]. Country- and production-specific factors may influence the occurrence of resistance [20, 21]. In our study, the patterns of resistance of most of the ESBL-*E. coli* isolates were either farm-specific or were shared by isolates from distant farms and distinct animal species. This observation suggests that ESBL-producing *E. coli* and their resistance profile spread within farms or arise independently.

None of the *E. coli* isolates were resistant to any of the quinolones tested (enrofloxacin, ciprofloxacin, nalidixic acid), probably reflecting the low use of these antibiotics in food-producing animals. A previous study showed a significant positive correlation between antibiotic dose and the occurrence of antibiotic-resistant bacteria in animal feces [22]. In our study, the discrepancy between the use of antimicrobials and the level of resistance is striking, as antimicrobial use was not always linked to AMR level, especially for β-lactams and tetracyclines. A potential bias might be underreported use of antimicrobials, which would explain the inverse relation, but there may be other reasons. The low level of resistance to tetracycline in beef cattle, despite the number of farms that used this drug, might be due to targeted treatment in small beef cattle production rather than the collective treatment used in larger-scale pig and poultry husbandry. Antimicrobial treatment also reflects the veterinary cost, which is lower for collective administration, e.g. to poultry. Collective treatment might therefore contribute to the emergence of resistance and should therefore be more closely controlled.

The high frequency of ESBL resistance observed in broilers (35.3%) and to a lesser extent in pig and beef cattle farms with no use of third-generation cephalosporins is difficult to explain. We tested imported 7-day old chicks 1 day after arrival from mainland France but found no resistance to these antimicrobials. It has been shown that a rapid increase in ESBL-*E. coli* prevalence in the first week of life must be due to factors other than latent contamination of the majority of birds at arrival [23]. Therefore, as no third-generation cephalosporins were administered in the production systems in which ESBL-*E. coli* resistance was detected, the observed resistance to these drugs was probably due to co-selection of several resistance genes in the same genetic determinant by other antibiotics, [24], but not specifically documented here. It has also been reported that tetracycline resistance in commensal *E. coli* is often linked to resistance to other antimicrobials, such as ampicillin and trimethoprim–sulfonamides [21]. A recent study on a Danish pig production farm showed clearly that commonly used antimicrobials such as tetracycline, which are not listed as critically important for human treatment, can promote resistance to critically important antimicrobials, limiting treatment possibilities [25]. A recent metagenomic study on bacterial communities showed that tetracycline resistance is often found in ESBL isolates and transmitted with ESBL-containing plasmids [26]. Moreover, an integrated approach to AMR found an increased prevalence of integrons containing resistance genes in tetracycline-resistant isolates [27]. The wide use of tetracyclines in Guadeloupe may explain some of the disproportion between the prevalence of resistance and the use of third-generation cephalosporins. These results reinforce the importance of animal food-producing systems as a reservoir of mobile genetic elements carrying multiple resistance determinants.

Further studies are warranted to better define the genetic background of ESBL-*E. coli* isolates and the context of AMR on Guadeloupe, especially in food-producing animals that are not exposed to third-generation cephalosporins.

## Conclusion

Our study provides the first baseline information on levels of antimicrobial use, on the dynamics of phenotypic and genotypic resistance to tetracyclines, and on ESBL-*E. coli* in small-scale pig, beef cattle, and poultry production on Guadeloupe. Despite rational use of antimicrobials, *E. coli* resistant to third-generation cephalosporins were found on the farms. Mechanisms other than selective pressure of these antimicrobials in the emergence of AMR remain to be elucidated.

## Methods

### Survey design

A prospective survey on the use of antimicrobial agents in veterinary medicine and food animal production was conducted between March and July 2018 on 14 pig, 16 beef cattle, and 15 poultry production farms. The farms were selected randomly in 16 of the 32 townships of the island to ensure representative production, covering small- to large-scale cooperative or independent production. All the pig facilities were farrow-to-finishing farms, with 30–3120 head per farm, for a total of 8549 pigs, representing 59.0% of pig production on Guadeloupe. Beef cattle breeding was investigated on 13 small-scale grassland farms (≤ 90 head) and three large-scale farms, for a total of 1691 head (mean age of 4.1 ± 2.4 years), representing 4.3% of local meat production. The poultry breeder farms had 400–64,000 birds, for a total of 184,510, representing 36.4% of local production [11].

Antimicrobial use was documented in declarative face-to-face interviews with farmers by an agronomist using a questionnaire specific for the study. Each participant provided information on farm characteristics (size, number of head) and routines for antimicrobial use, including frequency, reasons for treatment, name of the antibiotic drug used, route of administration, and estimated annual cost of treatment, including laboratory analyses, veterinary services, and drug purchase.

### Sampling and collection

Between January 2018 and May 2019, 11 pig farms, eight beef cattle farms, nine poultry farms, and the only slaughterhouse for beef cattle and pigs on Guadeloupe, representing respectively 24 and 10 farms, were screened for *E. coli*. As most small herds of beef cattle were raised free in fields, sampling was more difficult than that of pigs or poultry confined in blocks and is therefore less representative of the total production (4.3%) [11]. During the study, 216 fecal samples (30 g) were collected randomly just after excretion (124 from pigs of which 34 were slaughterhouse pigs and 75 from beef cattle of which 24 were slaughterhouse beef cattle). Fecal material from 17 hen houses was sampled by walking on litter approximately 100 m around a flock in boot socks (Sterisocks Tryptone SodiBox, Nevez, France). All samples were stored and transported in sterile cups or bags on ice to the Institut Pasteur laboratory within 4 h. Samples were stored at 4 °C and processed within 8 h of sampling.

### Bacterial isolation and identification

A 10-μL loop of each fecal sample was mixed in Luria Bertani broth BD Difco™ (Humeau, La Chapelle-sur-Erdre, France) supplemented or not with 4 mg/L of ceftriaxone and incubated at 37 °C for 24 h. Selective enrichment with 4 mg/L of ceftriaxone were streaked on chromogenic coliform agar plates (CHROMagar™, Paris, France) supplemented with 4 mg/L of ceftriaxone. Non-selective enrichments without 4 mg/L of ceftriaxone were streaked on chromogenic coliform agar plates without 4 mg/L of ceftriaxone. All plates were incubated at 37 °C for 24 h. One susceptible and three resistant metallic blue colonies were randomly picked up from non-selective and selective chromogenic coliform agar, respectively and identified by matrix-assisted laser desorption and ionization time-of-flight mass spectrometry on an Axima performance spectrometer (Shimadzu Corp, Osaka, Japan).

### Antimicrobial susceptibility analysis

The susceptibility of all *E. coli* isolates to 17 antimicrobials in six distinct classes was assessed in the standard disk diffusion method on Mueller-Hinton agar, as recommended [28]. Strains were tested against ampicillin (10 μg), amoxicillin–clavulanic acid (20 μg–10 μg), temocillin (30 μg), cefotaxime (5 μg), ceftazidime (10 μg), cefoxitin (30 μg), ertapenem (10 μg), gentamicin (10 μg), amikacin (30 μg), streptomycin (10 μg), enrofloxacin (5 μg), nalidixic acid (30 μg), ciprofloxacin (5 μg), tetracycline (30 IU), tigecycline (15 μg), trimethoprim–sulfamethoxazole (1.25 μg–23.75 μg), and fosfomycin (200 μg). ESBL producers were confirmed in the combined disk diffusion test with cefotaxime and ceftazidime with or without clavulanic acid. Growth inhibition diameters were measured with the Adagio™ automated system (Bio-Rad, Marnes-La-Coquette, France). *E. coli* strains were classified as susceptible, intermediate, or resistant according to the epidemiological thresholds [28] and intermediate isolates were classified as resistant for further analysis. *E. coli* ATCC 25922 was used as a quality control. *E. coli* with a similar AMR profile and isolated from the same sample were considered duplicates of the same clone and were counted only once.

### Resistance gene screening

For molecular characterization of ARG, genomic bacterial DNA was extracted from one colony with the InstaGene™ Matrix kit (Biorad, California, USA), according to the manufacturer’s instructions. ESBL and tetracycline resistance coding genes were screened by PCR in all *E. coli* tetracycline-resistant isolates. *bla*_CTX-M_ multiplex PCR including phylogenetic groups 1, 2, and 9 was performed [29]. *bla*_TEM_ *gene* was screened by simplex PCR [30]. Amplified PCR products were sequenced (Eurofins, Ivry-sur-Seine, France) and compared with known resistance gene sequences in the GenBank database by multiple-sequence alignment with the Basic Local Alignment Search Tool program for further characterization. Tetracycline-resistant isolates were screened for the presence of *tet*A and *tet*B genes with specific *tet*A primers designed for this study (*tet*A-F 5′-TAGAAGCCGCATAGATCGCC-3′ and *tet*A-R 5′-GCTTCATGAGCGCCTGTTTC-3′) and published specific *tet*B primers [31]. The duplex PCR amplification conditions for *tet*A and *tet*B were optimized as follow: 5 min at 95 °C, followed by 35 cycles at 95 °C for 30 s, at 62 °C for 30 s, and at 72 °C for 30 s, followed by a final extension at 72 °C for 7 min. The amplicons were detected by gel electrophoresis. For quality control, a subsample of 10% was genotyped twice.

### Combined numerical analysis

The combined numerical analysis was performed on ESBL-producing *E. coli* patterns with BioNumerics® v6.6 software (Applied Maths NV). Each file with experimental data from AMR and ARG screening was merged as a composite data set in the BioNumerics® database, with the similarity coefficient option taken from each experiment. The matrices from the individual experiments were averaged according to the same defined weight, and an individual similarity matrix was calculated in such a way that all characters had an equal influence on similarity. A dendrogram was drawn by using the unweighted pair group method with arithmetic averages with a tolerance of 1% to show the similarity of combined AMR and ARG patterns of the bacterial isolates.

### Statistical analysis

Results are presented as means ± standard deviation, medians with the interquartile ranges for quantitative variables, and numbers and percentages for qualitative variables. Intergroup differences among farms classified according to food-producing animal category were assessed with the Kruskal–Wallis or chi-square test, as appropriate. The level of significance was defined as *P* <  0.05. Analyses were conducted with STATA® 11.2.

## Data Availability

The datasets analyzed during the current study are available from the corresponding author on reasonable request.

## Abbreviations

AMR: Antimicrobial resistance
ESBL: Extended-spectrum β-lactamase
TET: Tetracycline
SM: Streptomycin
SXT: Trimethoprim–sulfamethoxazole
QNR: Quinolones
AMP: Ampicillin
NAL: Nalidixic acid
ARG: Antimicrobial resistance genes
PCR: Polymerase chain reaction
*iqr*: interquartile range
sd: standard deviation
